# Sensory-to-motor integration during auditory repetition: a combined fMRI and lesion study

**DOI:** 10.3389/fnhum.2014.00024

**Published:** 2014-01-31

**Authors:** ‘Ōiwi Parker Jones, Susan Prejawa, Thomas M. H. Hope, Marion Oberhuber, Mohamed L. Seghier, Alex P. Leff, David W. Green, Cathy J. Price

**Affiliations:** ^1^Wellcome Trust Centre for Neuroimaging, University College LondonLondon, UK; ^2^Wolfson College, University of OxfordOxford, UK; ^3^Institute of Cognitive Neuroscience, University College LondonLondon, UK; ^4^Cognitive, Perceptual and Brain Sciences, University College LondonLondon, UK

**Keywords:** fMRI, lesions, language, speech, aphasia

## Abstract

The aim of this paper was to investigate the neurological underpinnings of auditory-to-motor translation during auditory repetition of unfamiliar pseudowords. We tested two different hypotheses. First we used functional magnetic resonance imaging in 25 healthy subjects to determine whether a functionally defined area in the left temporo-parietal junction (TPJ), referred to as Sylvian-parietal-temporal region (Spt), reflected the demands on auditory-to-motor integration during the repetition of pseudowords relative to a semantically mediated nonverbal sound-naming task. The experiment also allowed us to test alternative accounts of Spt function, namely that Spt is involved in subvocal articulation or auditory processing that can be driven either bottom-up or top-down. The results did not provide convincing evidence that activation increased in either Spt or any other cortical area when non-semantic auditory inputs were being translated into motor outputs. Instead, the results were most consistent with Spt responding to bottom up or top down auditory processing, independent of the demands on auditory-to-motor integration. Second, we investigated the lesion sites in eight patients who had selective difficulties repeating heard words but with preserved word comprehension, picture naming and verbal fluency (i.e., conduction aphasia). All eight patients had white-matter tract damage in the vicinity of the arcuate fasciculus and only one of the eight patients had additional damage to the Spt region, defined functionally in our fMRI data. Our results are therefore most consistent with the neurological tradition that emphasizes the importance of the arcuate fasciculus in the non-semantic integration of auditory and motor speech processing.

## INTRODUCTION

Auditory repetition is a task that requires the immediate re-production of an auditory stimulus. This involves auditory processing of a heard sound, and then translation of the auditory input into an articulatory output that reproduces the sound of the original auditory input as closely as possible. This paper is concerned with the neurological underpinnings of this auditory-to-motor “translation,” “mapping,” or “integration,” process. At the cognitive processing level, we distinguish between semantically mediated and non-semantically mediated translation. Semantically mediated translation involves the production of speech from semantic representations, for example when naming the source of nonverbal sounds (e.g., “cat” in response to hearing a meow). Non-semantically mediated auditory-to-motor translation proceeds by prior learning of the mapping between auditory inputs and vocal tract gestures. This could be at the level of lexical representations (e.g., familiar words like “champion”), sublexical representations (e.g., sequences of syllables “cham-pi-on” or “cho-nam-pi” ) or non-verbal auditory features (e.g., when the human vocal tract is used to mimic nonverbal sounds that have neither phonological nor semantic associations). Here we are specifically interested in the translation of non-semantic auditory inputs to motor outputs.

With respect to the neural underpinnings of auditory-to-motor integration, the classic neurological model of language identifies Wernicke’s area (in the left posterior superior temporal cortex) as the site of “auditory images of speech” and Broca’s area (in the left posterior inferior frontal cortex) as the site of “motor images of speech,” with the arcuate fasciculus white-matter tract serving to integrate the auditory and motor images. According to this model, selective damage to the arcuate fasciculus that preserves Wernicke’s and Broca’s areas would impair auditory repetition in the context of intact speech comprehension and intact speech production (Geschwind, 1965). More recently, there have been claims that a cortical area on the left TPJ, known informally as sylvian-parietal-temporal (Spt), is actively involved in integrating auditory inputs with vocal tract gestures (Hickok et al., 2003; Hickok et al., 2009; Hickok, 2012). According to this perspective, selective deficits in auditory word repetition are the consequence of cortical damage to Spt (Buchsbaum et al., 2011). We examine this possibility in the context of functional magnetic resonance imaging (fMRI) and lesion studies, which allow us to examine auditory to motor translation. We start by considering prior functional imaging evidence for the functional role of Spt.

Sylvian-parietal-temporal region is functionally defined as an area at the posterior end of the lateral sulcus (Sylvian fissure), around the anterior end of the TPJ, which responds in general to both auditory perception and silent vocal tract gestures (Hickok et al., 2009; Hickok, 2012). For instance, Spt responds to covert rehearsal in tests of phonological short-term memory (Jacquemot and Scott, 2006; Koelsch et al., 2009). As Spt is involved in humming music and silent lip reading (Pa and Hickok, 2008; Hickok et al., 2009), it is not specific to speech input or output. Instead, the auditory-to-motor integration process has been described as a mechanism by which sensory information can be used to guide vocal tract action (Buchsbaum et al., 2011). Here we make a distinction between an area that acts as an interface between two tasks (i.e., a shared level of processing) and an area that is involved in integrating one level of processing with another. In other words, an interface region may be activated independently by separate tasks (logical OR), given that they share a common processing level, whereas an integration region should only be active when multiple processing levels are present (logical AND), and brought together (i.e., transformed) into an integrated output. If Spt is an integration area, rather than just an interface, then it should be more activated when the task involves the translation of sensory inputs to motor outputs. Previous studies have reported greater Spt activation for covert repetition than listening, and argued that this reflects the greater demands on auditory-to-motor integration during repetition (Isenberg et al., 2012). However, covert repetition may also increase the demands on subvocal articulation and auditory imagery of the spoken response (i.e., an internal representation of how the spoken response, or any other auditory stimulus, would sound). If Spt is involved in either of these processes (see below for evidence) then activation that is common to listening and covert repetition may reflect a shared level of processing rather than an active auditory-to-motor integration process. Prior to concluding that Spt actively integrates auditory information with motor output, we therefore need to factor out explanations that are related to subvocal articulation (independent of sensory input) or auditory processing (independent of motor output).

The association of TPJ with auditory processing and auditory imagery arose from early functional neuroimaging studies that observed left TPJ activation when subjects imagined hearing another person’s voice in the absence of any auditory stimulation or motor activity (McGuire et al., 1996). Subsequent studies have also shown left-lateralized activation in the TPJ in response to: silently imagining speech (Shergill et al., 2001); imagining the auditory relative to visual associations of a picture of a scene (Wheeler et al., 2000); experiencing tones and visual stimuli (Xue et al., 2006); silence following familiar music, even when there was no instruction to remember the music (Kraemer et al., 2005); passively viewing finger tapping on a piano following keyboard training (Hasegawa et al., 2004); producing rhythmic finger sequences that had been learnt with an auditory cue (Bengtsson et al., 2005); and imagining heard speech, music or environmental sounds in the absence of any acoustic stimulus (Aleman et al., 2005; Bunzeck et al., 2005; Zatorre and Halpern, 2005). Without a functional localizer it is unclear which, if any, of these responses in TPJ was generated in area Spt. Nevertheless, an explanation of Spt responses in terms of auditory imagery would explain the overlap of activation during auditory perception, subvocal articulation (Paus et al., 1996a,b; Wise et al., 2001), and silent auditory short-term memory tasks (Buchsbaum and D’Esposito, 2009; Koelsch et al., 2009; McGettigan et al., 2011) without the need to account for Spt activation in terms of a function that integrates auditory and motor processing.

The association of TPJ activation with subvocal articulation that occurs automatically during speech perception, particularly when speech perception is challenging (Buchsbaum and D’Esposito, 2009; Price, 2010), comes from observations that TPJ activation increased when subjects articulated four versus two syllables during a task that involved delayed repetition and subvocal rehearsal of pseudowords (Papoutsi et al., 2009). This subvocal articulation/articulatory rehearsal account can explain activation in TPJ during auditory working-memory tasks (Buchsbaum and D’Esposito, 2009; Koelsch et al., 2009) but does not explain why TPJ activation has been reported for auditory imagery of sounds that cannot be articulated (see above). It is therefore possible that different parts of TPJ are involved in auditory-to-motor integration, auditory imagery, and subvocal articulation. Our interest is in testing whether there is more evidence that Spt, located in TPJ, is involved in auditory motor integration than articulation or auditory processing alone.

Using fMRI, we defined the Spt area of interest functionally as being activated by both auditory speech perception and subvocal articulation (Hickok et al., 2003, 2009; Hickok, 2012). We then investigated whether any part of this Spt area was responsive to the demands on (1) non-semantic auditory-motor integration, (2) semantic to motor integration, (3) auditory input, and/or (4) articulation. By manipulating these factors independently, we aimed to determine the most likely level of processing that drives Spt. Our fMRI experiment (Paradigm 1) had 16 conditions in a 2 × 2 × 4 factorial design: auditory input versus visual input; speech production responses versus finger press responses; and four types of stimuli that weighted semantic and phonologically mediated speech production differentially. Moreover, to broaden our interpretation of Spt, we will also discuss the results of a second fMRI experiment (Paradigm 2) reported by Parker Jones et al. (2012). Without this second experiment, we could not rule out the possibility that an increased response in Spt merely reflected the integration of any sensory input and speech output, regardless of whether this integration was semantically mediated or not, as we explain below (see Materials and Methods).

In addition to investigating whether fMRI activation in Spt reflected the demands on auditory-to-motor integration, we also investigated lesion sites that were consistently associated with auditory repetition deficits in the context of intact word comprehension and production (i.e., conduction aphasia). Unlike a recent lesion study that looked for lesions associated with patients who had damage to both auditory repetition and picture naming (Buchsbaum et al., 2011), we were more interested in lesions that impaired auditory repetition while preserving the ability to name pictures. According to the neurological model, lesions associated with selective repetition difficulties were expected in the arcuate fasciculus, but according to functional neuroimaging data Spt involvement is also expected (Buchsbaum et al., 2011). We considered whether selective deficits in auditory repetition could occur following lesions to: (1) TPJ/Spt with minimal involvement of the underlying white matter; (2) the temporo-parietal white matter tracts (in the vicinity of the arcuate fasciculus) with minimal involvement of TPJ/Spt cortex; (3) both TPJ/Spt and the underlying white matter; and/or (4) neither TPJ/Spt nor the underlying white matter.

In summary, we used fMRI to test whether non-semantic auditory-to-motor translation during auditory repetition involved Spt or not, and then used lesion analyses to determine whether selective deficits in auditory repetition (i.e., conduction aphasia) were the consequence of lesions to Spt, the arcuate fasciculus, or both.

## MATERIALS AND METHODS

The study was approved by the London Queen Square Research Ethics Committee. All subjects gave written informed consent prior to scanning and received financial compensation for their time.

### FUNCTIONAL MAGNETIC RESONANCE IMAGING

#### Participants, fMRI Paradigm 1

In the fMRI study, the participants were 25 healthy, right-handed, native speakers of English, with normal or corrected-to-normal vision (12 females, 13 males, age range = 20–45 years, mean = 31.4 years, SD = 5.9 years). Handedness was assessed with the Edinburgh Handedness Inventory (Oldfield, 1971).

#### Experimental design, fMRI Paradigm 1

**FIGURE 1:**
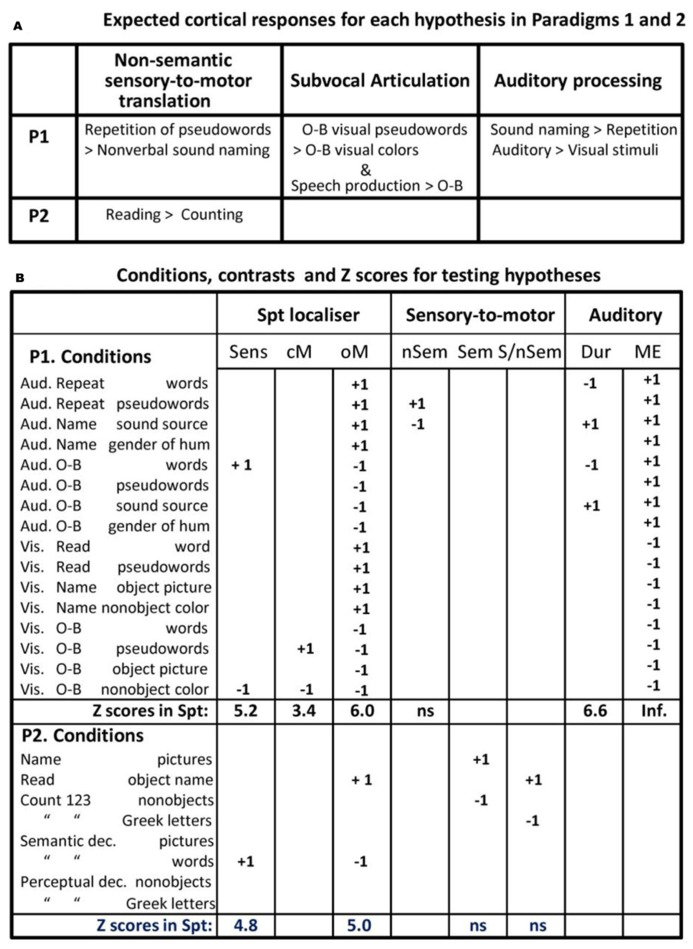
**Experimental hypothesis testing and results.** **(A**; top) describes the results that would support an interpretation of Spt activation in terms of sensory-to-motor integration, auditory imagery, and subvocal articulation. Note that the different accounts have opposing predictions for the same conditions (e.g., greater activation for pseudoword repetition than sound naming versus less activation for pseudoword repetition than sound naming). P1 = Paradigm 1, P2 = Paradigm 2 (see Materials and Methods). **(B**; bottom) lists the 16 different conditions, the statistical contrast used to test the different effects described in the top part of the figure, and the *Z* scores associated with each effect (i.e., the result). Aud = auditory presentation, Vis = visual presentation, O-B = one-back task, Articul. = Articulation, dec. = decision, Sens. = sensory speech input (no speech production), cM. = coverty mouth movements/articulation, oM. = overt mouth movements/articulation, nSem. = non-semantic, Sem. = semantic, S/nSem. = semantic and non-semantic, Dur. = auditory stimuli with long vs. short durations, ME. = main effect of auditory input, ns. = not significant.

The conditions of interest were auditory word and pseudoword repetition. However, these were embedded in a larger experimental design with a total of 16 different conditions (see **Figure 1B**) that allowed us to tease apart the activation related to auditory-to-motor translation from nonverbal auditory processing, auditory word perception, semantic processing, covert (subvocal) articulation, and overt articulation (see below for details).

The 16 conditions conformed to a 2 × 2 × 4 factorial design. Factor 1 was “stimulus modality”: auditory versus visual. Factor 2 was “task”: overt speech production in response to the stimulus versus one-back matching which involved a finger press response to indicate if the current stimulus was the same as the previous stimulus. Factor 3 was stimulus type, with four conditions that manipulated the presence or absence of phonological cues (i.e., words and pseudowords versus nonverbal stimuli) and the presence or absence of semantic stimuli (i.e., words, pictures, and nonverbal sounds of objects and animals versus pseudowords, meaningless scrambled pictures, and baseline stimuli). In the auditory modality, the stimuli were words, pseudowords, nonverbal environmental sounds, and humming in either a male or female voice. In the visual modality, the corresponding stimuli were words, pseudowords, pictures of objects, and pictures of scrambled objects.

In the speech production conditions, participants were instructed to: (a) repeat the auditory words and pseudowords which involves direct translation of auditory inputs to motor outputs; (b) name the source of the environmental sounds (e.g., “cat” in response to a meow), which involves semantically mediated auditory–motor translation; and (c) name the gender of the humming voice (male versus female), which served as the auditory baseline condition. The corresponding speech production conditions in the visual modality were: reading words and pseudowords (which involve direct visuo-motor translation); naming the objects in pictures (which involves semantically mediated visuo-motor translation); and naming the dominant color in meaningless pictures of nonobjects (the visual baseline condition).

In the eight silent one-back matching conditions (with exactly the same stimuli as the speech production conditions), participants were instructed to press a button box in response to each stimulus to indicate if the stimulus was the same or different to the previous one. Half the subjects used their right middle/index finger for the yes/no response. The other half used their left index/middle finger for the yes/no response. The proportion of repeated to non-repeated stimuli was 1:8. To keep the stimuli identical across tasks, stimuli were also repeated 1 every eight trials in the speech production conditions.

#### Stimulus selection/creation, fMRI Paradigm 1

Stimulus selection started by generating 128 pictures of easily recognizable animals and objects (e.g., cow, bus, elephant, plate) with one to four syllables (mean = 1.59; SD = 0.73). Visual word stimuli were the written names of the 128 objects, with 3–12 letters (mean = five letters; SD = 1.8). Auditory word stimuli were the spoken names of the 128 objects (mean duration = 0.64 s; SD = 0.1), recorded by a native speaker of English with a Southern British accent approximating Received Pronunciation. Pseudowords were created using a non-word generator (Duyck et al., 2004) and matched to the real words for bigram frequency, number of orthographic neighbors, and word length. The same male speaker recorded the auditory words and pseudowords.

The nonverbal sounds associated with objects were available and easily recognizable for a quarter (i.e., 32) of the stimuli, and taken from the NESSTI sound library (http://www.imaging.org.au/Nessti; Hocking et al., 2013). The duration of the nonverbal sounds needed to be significantly longer (mean length = 1.47 s, SD = 0.13) than the duration of the words (*t* = 37.8; *p* < 0.001) because shorter sounds were not recognizable. The auditory baseline stimuli were recorded by male and female voices humming novel pseudowords, thereby removing any phonological or semantic content (mean length = 1.04 s, SD = 0.43). Half of these stimuli were matched to the length of the auditory words, the other half to the length of the nonverbal sounds. The visual baseline stimuli were meaningless object pictures, created by scrambling both global and local features, and then manually edited to accentuate one of eight colors (brown, blue, orange, red, yellow, pink, purple, and green). Consistent speech production responses were ensured for all stimuli in a pilot study conducted on 19 participants.

#### Stimulus and task counterbalancing, fMRI Paradigm 1

The 128 object stimuli were divided into four sets of 32 (A, B, C, and D). Set D was always presented as nonverbal sounds. Sets A, B, and C were rotated across pictures, visual words, and auditory words in different participants. All items were therefore novel on first presentation of each stimulus type (for task 1) and the same items were repeated for task 2. Half of the subjects performed all eight speech production tasks first (task 1) followed by all eight one-back tasks (task 2). The other half performed all eight one-back tasks first (task 1) followed by all eight speech production tasks (task 2). Within each task, half of the subjects were presented auditory stimuli first, followed by visual stimuli; the other half were presented visual stimulus first, followed by auditory stimuli. The order of the four stimulus types was fully counterbalanced across subjects, and full counterbalancing was achieved with 24 participants.

Each set of 32 items was split into four blocks of eight stimuli, with one of the eight stimuli repeated in each block to make a total of nine stimuli per block (eight novel, one repeat). The stimulus repeat only needed to be detected and responded to (with a finger press) in the one-back tasks.

#### Data acquisition, fMRI Paradigm 1

Functional and anatomical data were collected on a 3T scanner (Trio, Siemens, Erlangen, Germany) using a 12-channel head coil. Functional images consisted of a gradient-echo EPI sequence and 3 mm × 3 mm in-plane resolution (TR/TE/flip angle = 3080 ms/30 ms/90°, EFOV = 192 mm, matrix size = 64 × 64, 44 slices, slice thickness = 2 mm, interslice gap = 1 mm, 62 image volumes per time series, including five “dummies” to allow for T1 equilibration effects). The TR was chosen to maximize whole brain coverage (44 slices) and to ensure that slice acquisition and stimulus onsets were a synchronized, which allowed for distributed sampling of slice acquisition across the study (Veltman et al., 2002).

For anatomical reference, a T1 weighted structural image was acquired after completing the tasks using a three-dimensional modified driven equilibrium Fourier transform (MDEFT) sequence (TR/TE/TI = 7.92/2.48/910 ms, flip angle = 16°, 176 slices, voxel size = 1 mm × 1 mm × 1 mm). The total scanning time was approximately 1 h and 20 min per subject, including set-up and the acquisition of an anatomical scan.

#### Procedure, fMRI Paradigm 1

Prior to scanning, each participant was trained on all tasks using a separate set of all training stimuli except for the environmental sounds which remained the same throughout both training and experiment. All speaking tasks required the subject to respond verbally by saying a single object name, color name or pseudoword after each stimulus presentation, whereas the one-back matching task required a button press (and no speech) after each stimulus presentation to indicate whether the stimulus was identical to the one immediately preceding it (yes with one finger/no with another finger). All participants were instructed to keep their body and head as still as possible and to keep their eyes open throughout the experiment and attend to a fixation cross on screen while listening to the auditory stimuli. Each of the 16 tasks was presented in a separate scan run, all of which were identical in structure.

Scanning started with the instructions “Get Ready” written on the in-scanner screen while five dummy scans were collected. This was followed by four blocks of stimuli (nine stimuli per block, 2.52 s inter-stimulus-interval, 16 s fixation between blocks, total run length = 3.2 min). Every stimulus block was preceded by a written instruction slide (e.g., “Repeat”), lasting 3.08 s each, which indicated the start of a new block and reminded subjects of the task. Visual stimuli were each displayed for 1.5 s. The pictures subtended an angle of 7.4° (10 cm on screen, 78 cm viewing distance) with a pixel size of 350 × 350, with a screen resolution of 1024 × 768. The visual angle for the written words ranged from 1.47° to 4.41° with the majority of words (with five letters) extending 1.84°–2.2°.The length of sound files varied across stimuli and tasks, ranging from 0.64 to 1.69 s (see stimulus creation above). Auditory stimuli were presented via MRI compatible headphones (MR Confon, Magdeburg, Germany), which filtered ambient in-scanner noise. Volume levels were adjusted for each subject before scanning. Each subject’s spoken responses were recorded via a noise-cancelling MRI microphone (FOMRI IIITM Optoacoustics, Or-Yehuda, Israel), and transcribed manually for off-line analysis. We used eye-tracking to ensure participants paid constant attention throughout the experiment.

#### Data Pre-processing, fMRI Paradigm 1

We performed fMRI data preprocessing and statistical analysis in SPM12 (Wellcome Trust Centre for Neuroimaging, London, UK), running on MATLAB 2012a (Mathsworks, Sherbon, MA, USA). Functional volumes were (a) spatially realigned to the first EPI volume and (b) un-warped to compensate for non-linear distortions caused by head movement or magnetic field in homogeneity. The anatomical T1 image was (c) co-registered to the mean EPI image which had been generated during the realignment step and then spatially normalized to the Montreal Neurological Institute (MNI) space using the new unified normalization-segmentation tool of SPM12. To spatially normalize all EPI scans to MNI space, (d) we applied the deformation field parameters that were obtained during the normalization of the anatomical T1 image. The original resolution of the different images was maintained during normalization (voxel size 1 mm × 1 mm × 1 mm for structural T1 and 3 mm × 3 mm × 3 mm for EPI images). After the normalization procedure, (e) functional images were spatially smoothed with a 6 mm full-width-half-maximum isotropic Gaussian Kernel to compensate for residual anatomical variability and to permit application of Gaussian random-field theory for statistical inference (Friston et al., 1995).

In the first-level statistical analyses, each pre-processed functional volume was entered into a subject specific, fixed-effect analysis using the general linear model (Friston et al., 1995). All stimulus onset times were modeled as single events, with two regressors per run, one modeling instructions and the other modeling all stimuli of interest (including both the repeated and unrepeated items). Stimulus functions were then convolved with a canonical hemodynamic response function. To exclude low-frequency confounds, the data were high-pass filtered using a set of discrete cosine basis functions with a cut-off period of 128 s. The contrasts of interest were generated for each of the 16 conditions of interest (relative to fixation).

#### Effects of interest, fMRI Paradigm 1

At the second level, the 16 contrasts for each subject were entered into a within-subject, one-way ANOVA in SPM12. From this analysis, we identified activation that increased in conditions that we hypothesized to tap the processing type of interest. A summary of the condition comparisons used to test our main hypotheses is provided in **Figure 1**. As with all imaging studies, the task analysis (i.e., the functional sub-processing involved in each task) involves a certain degree of *a priori* assumptions. Below, we try to make these assumptions and their bases explicit as well as testing their validity within the available data.

The effect of most interest was the location of activation associated with the non-semantic translation of auditory inputs to motor outputs. This was defined, *a priori*, as the area(s) where activation increased for repeating auditory pseudowords (that links auditory inputs to articulatory outputs) compared to naming nonverbal sounds (that accesses articulatory outputs from semantics). To control for auditory speech processing that is not integrated with a motor response, we also computed the interaction between stimulus (pseudowords > nonverbal sounds) and task (speech production that links the stimuli to articulation versus one-back matching that links the stimuli to a finger press response).

### DEFINING OUR REGION OF INTEREST IN Spt/TPJ

In addition to conducting a whole brain search for areas that were more activated for pseudoword repetition than nonverbal sound naming, we also conducted a region of interest analysis, with a small volume FWE correction for multiple comparisons, focusing on the Spt area associated with sensory-motor integration in Hickok and Poeppel (2007), Hickok et al. (2009), and Hickok (2012) who define Spt functionally as an area at the posterior end of the lateral sulcus (Sylvian fissure), around the anterior end of the TPJ, which responds to both auditory perception and silent vocal tract gestures (=subvocal articulation during speech tasks). We used the same functional definition, locating Spt in TPJ where activation increased during (a) auditory word perception, (b) covert (subvocal) articulation, and (c) overt speech production–with the assumption that areas associated with covert speech production should also be activated during overt speech production.

Areas associated with auditory word perception, when motor output was controlled, were identified by comparing activation for (a) one-back matching on auditory words and (b) one-back matching oncolors. Areas associated with subvocal articulation, were identified by comparing activation for (a) one-back matching on visual pseudowords and (b) one-back matching oncolors. Areas associated with overt speech production were identified by comparing all eight speech production conditions to all eight one-back matching conditions. See **Figure 1B** for summary.

Our reasons for using visual pseudoword matching to identify areas involved in subvocal articulation were fourfold. First, on the basis of cognitive processing models of reading (e.g., Seidenberg and McClelland, 1989; Coltheart et al., 1993), we hypothesized that accurate one-back matching on visually presented pseudowords could either be based on orthographic similarity or phonological similarity. Second, we hypothesized that phonological processing of orthographic inputs involves subvocal articulatory activity related to how the sounds associated with the inputs would be produced by the motor system. This hypothesis was based on prior work showing that articulatory areas are activated in response to visual pseudowords even when participants are performing an incidental visual matching task (see, Price et al., 1996). Third, evidence for articulatory processing during one-back matching of visual pseudowords in the current paradigm comes from the observation that a left premotor area (at MNI co-ordinates *x* = -51, *y* = -3, *z* = +33) is activated for the one-back task on pseudowords > words (*Z* score = 3.65), and, in turn, this region is activated during overt articulation (i.e., a main effect of speech > one-back tasks; *Z* score = 6.7). Thus, one-back matching on visually presented pseudowords covertly increased activation in areas, that are undisputedly associated with overt articulation, even though no overt articulation was involved. Fourth, by ensuring that our Spt area also responded to overt speech production, irrespective of stimulus type, we hypothesized that overlapping activation during silent one-back matching on visually presented pseudowords was more likely to be related to subvocal articulation than orthographic processing.

Consistent with the above hypotheses, we found activation (significant at *p* < 0.001 uncorrected) in TPJ for (i) one-back matching of auditory words relative to colors, (ii) one-back matching on visual pseudowords relative to colors, and (iii) all eight overt speech production conditions relative to all eight one-back matching conditions. The peak of this effect in MNI co-ordinates [-51, -39, +21] corresponds closely to the location of the Spt area reported by Hickok et al. (2009) where the mean effect across multiple single subjects analyses was located at Talairach co-ordinates [-50, -40, +19] which is [-51, -42, +18] in MNI space. As in our study, the Spt activation reported in Hickok et al. (2009) cannot be related to orthographic processing because it was identified using auditory stimuli only. Specifically, Hickok et al. (2009) identified activation related to covert articulation by comparing (a) a condition where participants hear speech and then covertly rehearse it to (b) a baseline condition where participants hear speech without instructions to covertly rehearse it.

In short, our definition of Spt was consistent with prior studies. Therefore our Spt-ROI for paradigm 1 was defined as the 33 contiguous voxels [around MNI co-ordinates (-51, -39,+21)] that were significant at *p* < 0.001 for (a) one-back matching on auditory words > colors, (b) one-back matching on visually presented pseudowords > colors, and (c) all overt speech production conditions relative to all one-back matching conditions.

### EXPLORING THE RESPONSE IN OUR FUNCTIONALLY DEFINED Spt AREA

After defining our Spt region of interest, and testing whether it was involved in non-semantic auditory to motor translation (i.e., for auditory repetition of pseudowords more than nonverbal sound naming), we also tested whether our Spt area was sensitive to auditory processing, when articulatory processing was controlled. We dissociated auditory processing and articulatory processing by comparing activation for overtly articulating animal and object names during (a) the nonverbal sound naming conditions (say “cat” when hearing a meow) and (b) the auditory word repetition conditions (say “cat” when hearing “cat”). Activation in auditory processing areas was expected to be higher for hearing nonverbal sounds than auditory words because the duration of all the nonverbal sound stimuli (mean = 1.47 s, SD = 0.13) was significantly longer (*t* = 37.8; *p* < 0.001) than the duration of all the word stimuli (mean = 0.64 s; SD = 0.1). We also expected that, if our Spt area was sensitive changes in early auditory processing, then its response across conditions should mirror that seen in the early auditory cortex (e.g., Heschl’s gyrus) and be greater during the auditory conditions than the corresponding visual conditions.

#### Additional functional data, fMRI Paradigm 2

In the fMRI design described above (Paradigm 1), all our speech production conditions involved the translation of sensory inputs to motor outputs in so far as the speech production output depended on the content of the sensory input. Therefore, as noted in the Introduction, we cannot fully exclude the possibility that an increased Spt response for speech production relative to one-back matching reflected the translation of any type of sensory input to speech outputs, irrespective of whether the sensory-to-motor translation was semantically or non-semantically mediated. We therefore report one further result from Parker Jones et al. (2012) The results we report were based on 36 native (monolingual) speakers of English. Full details of this second experimental paradigm, can be found in Parker Jones et al. (2011). In brief, Paradigm 2 included eight different conditions that involved either speech production, semantic matching, or perceptual matching (PM) on four types of stimuli (pictures of familiar objects, written names of the same familiar objects, pictures of meaningless non-objects and meaningless strings of Greek letters), see **Figure 1B** for a list of the eight conditions.

The result of interest in Paradigm 2 concerned the level of Spt activation for two conditions that require speech production in response to sensory input (overt picture naming and reading) relative to two conditions that do not involve sensory-to-motor translation (saying “1-2-3” repeatedly to meaningless visual cues). In other words, if Spt is involved in semantically and non-semantically mediated sensory-to-motor integration then activation in Spt should be higher for naming and reading than repeatedly saying “1-2-3,” irrespective of the visual input.

For this paradigm, we functionally defined Spt where there was an overlap of activation, in the TPJ territory, for (a) silent semantic decisions on written words relative to fixation (*p* < 0.001 uncorrected) and (b) reading aloud relative to semantic decisions on the same words (*p* < 0.001 uncorrected). The former contrast tapped word comprehension, the latter contrast involved overt speech production. The peak MNI co-ordinates for the overlapping activation were identified in TPJ at [-54, -38, +22] with a second peak at [-56, -42, +18]. Both peaks overlap with the P1-Spt-ROI. All surrounding contiguous voxels that were significant at *p* < 0.001 for both (a) and (b) were saved as the P2-Spt-ROI.

### LESION STUDY

#### Patient selection

Eight patients with selective deficits in auditory repetition were selected from the PLORAS database (Price et al., 2010) which includes lesion images and behavioral data from the Comprehensive Aphasia Test (CAT; Swinburn et al., 2004) and a continuously increasing population of Stroke patients (Price et al., 2010). The heterogeneity of patients in the database allows us to carefully select subsamples that are closely matched for all but one factor of interest. Patients are only excluded from this database if they have other neurological or psychiatric conditions, are unable to tolerate 2 h of speech and language assessments, or have implants or other contraindications to MRI scanning.

A neurologist (co-author Alex P. Leff) recorded whether the stroke resulted in left hemisphere, right hemisphere, or bilateral damage, and provided a comprehensive description of the lesion location. In addition, the lesion in each MRI scan was identified automatically as detailed below.

For the current study, we selected patients who were assessed 1–10 years after a left hemisphere stroke (ischemic or haemorrhagic) in adulthood (age range = 18–87 years), were right handed prior to their stroke, with English as their first language and with complete behavioral data on the CAT, and had focal lesions that were 50 cm^3^ or less. They were assessed on auditory repetition of words and non-words (pseudowords), picture naming, verbal fluency, auditory and written word comprehension, and semantic picture matching as described below. The inclusion criteria were scores in the aphasic range for word or pseudoword repetition and scores in the non-aphasic range for all other tasks picture naming, verbal fluency, auditory and written word comprehension, and semantic picture matching.

***Auditory word repetition.*** This required an immediate response to each heard word, presented one at a time. There were 16 words with 1–3 syllables. Correct responses were given a score of 2 if promptly produced, and 1 if production was accurate but delayed (>5 s) or if a self-correction or if a repetition of the stimulus was required. There were no points for absent or incorrect responses, including “phonemic” (i.e., segmental), neologistic, and dyspraxic errors. Dysarthric errors were not penalized. We selected patients whose *t*-value was 52 or less (see **Table 1**), thereby excluding patients who had normal or mildly aphasic auditory word repetition.

**Table 1:** Patients with conduction aphasia.

Patient number:	PS401	PS518	PS040	PS180	PS163	PS597	PS091	PS074
Repetition W	51	52	52	46	49	51	49	65
Repetition NW	53	53	53	53	51	49	67	49
Picture naming	64	74	74	64	64	66	64	74
Fluency	59	75	64	62	70	71	75	70
Aud comp W	65	65	53	55	55	53	65	58
Vis comp W	59	65	65	59	65	59	55	59
Age (years)	60.9	62.2	44.2	69.5	46.8	41.6	68.6	66.0
Years since stroke	5.5	3.5	2.8	1.6	1.3	4.8	5.8	2.0
Lesion volume (cm^3^)	50.4	17.1	20.6	25.7	31.3	34.9	38	5.9
Gender	F	M	F	M	F	M	M	M

*The results of the Comprehensive Aphasia Test (CAT) used to select the eight conduction aphasics are presented along with their age, years since stroke, lesion volume, and gender (see Materials and Methods). For each of the CAT assessments, *t*-values provide a standardized metric of abnormality (the position that a patient would have relative to a population of aphasics) rather than performance *per se*. These *t*-values therefore account for the fact that different assessments are not all equally difficult (Swinburn et al., 2004; p. 103). Abnormally low scores on the auditory repetition tasks are highlighted in dark gray. Scores that are on the border of normal/abnormally low are highlighted in light gray. Patient numbers (e.g., PS401) correspond to those from the PLORAS database (Price et al., 2010). W = words, NW = nonwords = pseudowords, Aud = Auditory, Vis = Visual, Comp = comprehension, F = female, M = male.*

***Auditory non-word repetition.*** Auditory repetition of five heard non-words (syllable range 1–2). Scoring was as for word repetition. Unlike word repetition, repetition of non-words cannot be facilitated by word recognition or semantic processing; it is entirely reliant on phonological processing. The memory load may therefore be higher than that required for auditory word repetition. We selected patients whose *t*-value was 52 or less (see **Table 1**), thereby excluding patients who had normal or mildly aphasic auditory word repetition.

***Picture naming.*** Patients were asked to generate the names of objects or animals in response to 24 black-and-white line drawings presented one at a time. Correct items, were given a score of 2 if accurate and promptly named, and 1 if accurate but delayed (>5 s) or if a self-correction. Incorrect responses or responses only obtained after a semantic and/or phonological cue were given a score of zero. We excluded patients who had either mildly or severely aphasic responses.

***Verbal fluency.*** This score is a sum of two component tests: category fluency (“Name as many animals as you can”) and phonological fluency (“Name words beginning with the letter ‘s’ ”). Each subject was allowed 60 s for each test. Subjects were allowed to make articulatory errors but repeated items (perseverations) were not counted. There was no auditory perceptual component to this task (other than self-monitoring). It was designed primarily to test word retrieval and is commonly used as a test of central executive processing (Baddeley, 1996). In this paper, we report a composite measure of semantic and phonological fluency and excluded patients who had either mildly or severely aphasic scores.

***Single-word auditory comprehension.*** Subjects were presented with four black-and-white line drawings and a spoken word was presented. Subjects had to point to the corresponding target drawing. Alongside the target drawing there were three distractors. One was phonologically related to the target, one was semantically related, and one was unrelated. Subjects could request that the word was repeated without penalty. Subjects scored one point if they pointed to the correct target. There were 15 presentations in total. We excluded patients who had either mildly or severely aphasic responses.

***Single-word visual comprehension.*** This subtest is constructed along the same lines as the single-word auditory comprehension test above except that the phonological distracters are both phonologically and *visually* similar to the target when the words are written down (e.g., target: “pin”; distractors: “bin,” “needle,” “basket”). The rated semantic similarity of target and semantic distractor is equal in the two subtests, allowing a direct comparison of the relative degree of impairment in the auditory and visual word comprehension. Different words were used in the auditory and visual versions of the task. We excluded patients who had either mildly or severely aphasic responses.

***Semantic memory.*** The task involved visual presentation of an image in the center of a page surrounded by four other images. All images were black and white line drawings. Patients were instructed to point to the drawing that “goes best with,” i.e., is most closely semantically related to the target object (e.g., hand). One of the four drawings was a good semantic match to the target (e.g., mitten), one was a close semantic distractor (e.g., sock), one more distantly related (e.g., jersey), and one was unrelated (e.g., lighthouse). One mark was awarded for each correct response. Successful performance on this task indicated that the patient had recognized the picture and accessed detailed semantic associations. We excluded patients who had either mildly or severely aphasic responses.

Images acquired from our Siemens 1.5 T Sonata (*n* = 5) had an image matrix of 256 × 224, with repetition time/echo time = 12.24/3.56 ms. Images acquired from our Siemens 3T Trio scanners had an image matrix of 256 × 256 (*n* = 2), with repetition time/echo time = 7.92/2.48 ms. Images acquired from our Siemens 3T Allegra (*n* = 1) had an image matrix of 256 mm × 240 mm, with repetition time/echo time = 7.92/2.4/530 ms.

The lesions were identified from the anatomical MRI images using a fully automated procedure described in Seghier and Price (2013). In brief, scans were pre-processed in SPM5/8 (Wellcome Trust Centre for Neuroimaging, London, UK), with spatial normalization into standard MNI space using a modified implementation of the unified segmentation algorithm that was optimized for use in patients with focal brain lesions. After segmentation and normalization, gray and white matter tissue images were smoothed and subsequently compared to control data from 64 healthy subjects. This identified abnormal voxels using an outlier detection algorithm that generates a binary image of the lesion site in standard MNI space (Seghier et al., 2008). Abnormal voxels in gray and white matter were finally grouped and delineated as lesions, creating a three-dimensional image of individual patients’ lesions in MNI space. Individual lesions were then overlaid to create 3D lesion overlap maps, showing where patients shared damage at each voxel of the brain.

## RESULTS

### IN-SCANNER BEHAVIOR

**FIGURE 2:**
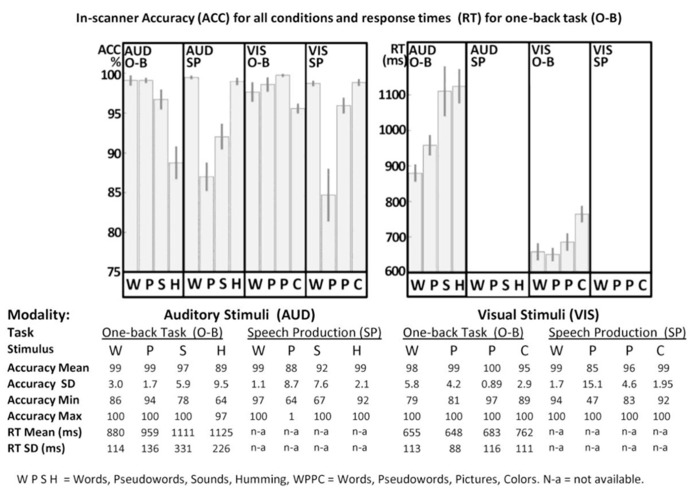
**In-scanner performance.** Accuracy (ACC) and response times (RT) for one-back (O-B) and speech production (SP) tasks are plotted in the top part of the figure for both visual (VIS) and auditory (AUD) modalities, where error-bars represent standard errors. Full details are provided in the bottom part of the figure. WPSH = words, pseudowords, sounds and humming. WPPC = words, pseudowords, pictures and colors. SD = standard deviation, Min = minimum, Max = maximum, n-a = not available. For technical reasons, data for three participants were excluded from all O-B tasks.

Details of the in-scanner behavior are provided in **Figure 2**. Statistical analyses involved 2 × 4 ANOVAs in SPSS manipulating stimulus modality (visual versus auditory) with stimulus type (word, pseudoword, sound/picture, and gender/color). All ANOVAs were corrected for potential violations of sphericity, adjusting their degrees of freedom using the Greenhouse–Geisser correction (Greenhouse and Geisser, 1959). These corrections result in more conservative statistical tests (i.e., decreasing the risk of false positives while increasing the risk of false negatives), and account for the non-integer degrees of freedom below. Data from all 25 subjects were included for the speech production tasks (measuring accuracy in both visual and auditory modalities), while data from only 22 subjects were included for the one-back tasks [measuring accuracy and response times (RT) in both visual and auditory modalities]. Three subjects’ data were lost in the one-back tasks for technical reasons.

For speech production accuracy, we found a main effect across the four stimulus type conditions [*F*(1.38,33.11) = 29.14; *p* < 0.001, Greenhouse–Geisser] and a stimulus modality by condition interaction [*F*(1.52,36.41) = 3.82; *p* = 0.042, Greenhouse–Geisser] but no overall effect of stimulus modality [*F*(1.00,24.00) = 0.04; *p* = 0.84, Greenhouse–Geisser]. In the visual domain, accuracy was higher for words and colors than pictures and pseudowords. In the auditory domain, accuracy was higher for words and gender than sounds or pseudowords. Response time data were not available in the speech production task.

For accuracy in the one-back task (with partially missing data for three subjects), we found a main effect across the four stimulus type conditions [*F*(2.25,47.32) = 29.94; *p* < 0.001, Greenhouse–Geisser], a main effect of stimulus modality [*F*(1.00,21.00) = 4.89; *p* = 0.038, Greenhouse–Geisser] and a stimulus modality by condition interaction [*F*(2.08,43.65) = 6.54; *p* = 0.003, Greenhouse–Geisser]. In the visual domain, accuracy was higher for pictures, pseudowords and words relative to colors. Likewise, in the auditory domain, accuracy was higher for words, pseudowords and sounds than gender. The lower accuracy for color and gender arose because some participants attempted to match these stimuli on their visual or auditory forms, rather than their color or pitch.

For RT in the one-back task, we found a main effect across the four stimulus type conditions [*F*(1.62,34.07) = 21.17; *p* < 0.001, Greenhouse–Geisser], a main effect of stimulus modality [*F*(1.00,21.00) = 150.51; *p* < 0.001, Greenhouse–Geisser], and a stimulus modality by condition interaction [*F*(1.81,38.00) = 6.68; *p* = 0.004, Greenhouse–Geisser]. For all conditions, participants were slower in the auditory modality than the visual modality. Within both stimulus modalities, RT mirrored the accuracy on the one-back task with faster response time and higher accuracy for words and pseudo-words compared to the baseline conditions (gender and color).

### fMRI RESULTS

#### Non-semantic auditory-to-motor translation, fMRI Paradigm 1

No brain areas, including Spt, were more activated by auditory repetition of pseudowords compared to sound naming. At the individual subject level, only one subject showed higher activation for pseudoword repetition than sound naming but this did not approach significance (MNI *x* = -51, *y* = -45, *z* = +15; *Z* score = 2.1; *p* > 0.05 following small volume correction for multiple comparisons). This null result leaves us with two questions: (1) is auditory-to-motor translation a function of the white matter connections (see lesion analysis below) and (2) what is the function of Spt in the TPJ.

#### Auditory activation in area Spt, fMRI Paradigm 1

**FIGURE 3:**
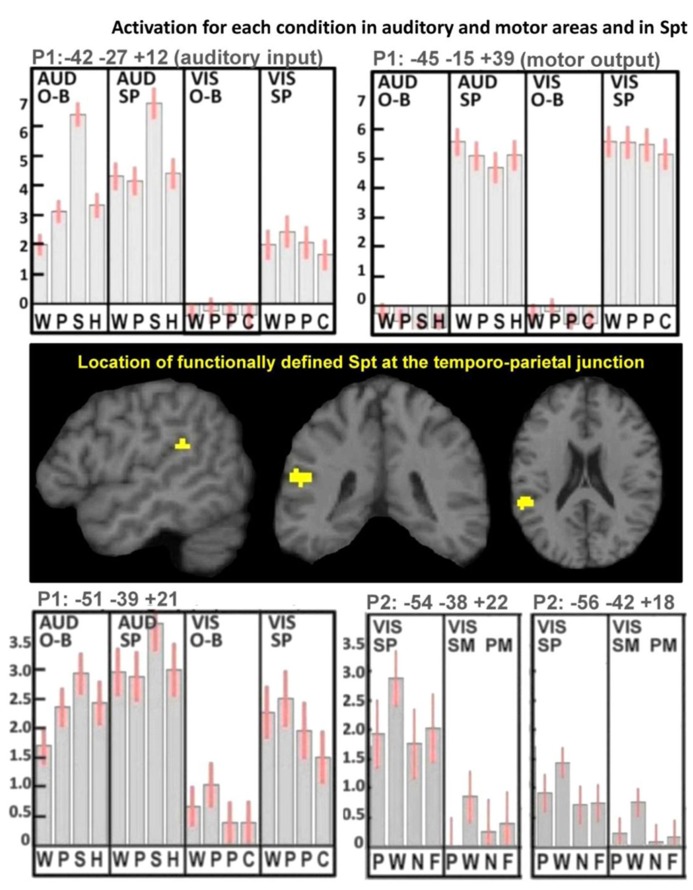
**Activation for each condition in auditory and motor areas, and in Spt.** These results illustrate the mean activation responses across all conditions in primary auditory and motor areas as well as in Spt. The top plots show activation responses for one-back (O-B) and speech production (SP) tasks in both auditory (AUD) and visual (VIS) modalities in left Heschl’s gyrus (top-left plot, labeled “auditory input”) and left central sulcus (top-right plot, labeled “motor output”). In the AUD modality, the stimuli were words, pseudowords, environmental sounds, and humming (WPSH). In the VIS modality, the stimuli were words, pseudowords, pictures of objects, and pictures of scrambled objects (WPPC). The center images locate our functionally defined mask for Spt at the TPJ. The bottom plots show activation responses in Spt in Paradigm 1 (P1; bottom-left plot) and in Paradigm 2 (P2; bottom-right plot). As both top plots use P1, the conditions are the same in the bottom-left plot. The bottom-right plot shows the primary and secondary peaks for Spt in P2, where the tasks were spoken response (SP), semantic matching (SM), and perceptual matching (PM) all in the visual (VIS) modality. Stimuli comprised pictures, words, nonobjects, and false-fonts (PWNF). In all five plots, error bars represent 90% confidence intervals. See Section “Materials and Methods.” Note that in P1, activation in both Heschl’s gyrus and Spt is lowest for the visual one-back task (O-B) because there was no auditory input in either the stimulus or the response. During the visual speech production conditions (VIS-SP) activation was observed in auditory areas because participants can hear the sound of their spoken response.

There were highly significant increases in Spt activation when auditory input increased (see **Figure 1B**). Specifically, (1) Spt activation was higher (*Z* score = 6.6) for hearing and responding to nonverbal sounds of objects and animals than their heard names which had less than half the auditory duration of the sounds (mean 1.47 vs. 0.64 s, *t* = 37.8, *p* < 0.001); and (2) Spt activation was higher (*Z* score = 6.7) for the direct comparison of all auditory stimuli to all visual stimuli. A third relevant observation, illustrated in **Figure 3**, is that the pattern of activation in Spt over the eight auditory conditions mirrored that seen in Heschl’s gyrus and the primary auditory cortex [compare the plot at (-51, -39,+21) and (-42, -27,+12)].

#### Other types of sensory to motor activation in area Spt, fMRI Paradigm 2

Activation in the P2-Spt-ROI, was greatest for reading aloud but did not differ for object naming (semantically mediated sensory-to-motor translation) and repeatedly saying “1-2-3” (no sensory-to-motor translation); see lower right-hand corner in **Figure 3**. Therefore we found no evidence that Spt was involved in either semantic or non-semantically mediated sensory to motor translation.

### LESIONS RESULTING IN SELECTIVE AUDITORY REPETITION DEFICITS

**FIGURE 4:**
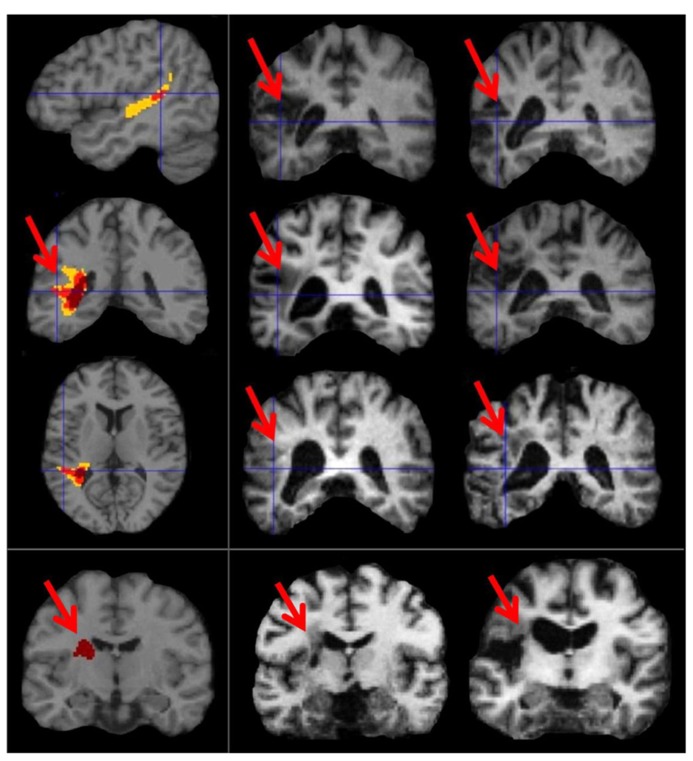
**Lesion sites in patients with selective repetition difficulties.** These images illustrate the most consistent lesion sites in patients with selective repetition difficulties. The left column shows overlap maps. The first three rows of the left column show overlap maps of sagittal (*x* = -40), coronal (*y* = -40), and axial slices (*z* = +10) for six patients projected onto the canonical brain in MNI space. To the right, these six patients are represented by coronal sections of their individual anatomical brain images in normalized space (in the middle and right columns of rows 1, 2, and 3). The bottom row shows the coronal overlap map (left column) for two patients (middle and right columns). These bottom two patients (PS091 and PS597) show lesions more anterior (*y* = -15) to the six patients above (*y* = -40). In the top three overlap maps, yellow indicates a lesion overlap of 4/6 patients, red a lesion overlap of 5/6 patients, and dark maroon a lesion overlap of 6/6 patients (i.e., the maximum possible overlap). In the bottom overlap map, the maximum overlap is 2/2 patients, which is indicated again by dark maroon. Red arrows point to the area of overlap in each patient and in the coronal overlap maps.

At the time of analysis (May, 2013), eight patients in the PLORAS database met our inclusion criteria (see **Table 1** for details). The lesion overlap map (**Figure 4**) shows that six of the patient patients had damage to the temporo-parietal component of the superior longitudinal fasciculus, corresponding to the location of the arcuate fasciculus. The lesion extended ventrally, undercutting the left posterior superior temporal area (*z* = +8 in MNI space) associated with phonological processing during both speech perception and production (Wise et al., 2001). This is illustrated in **Figure 4** by showing sagittal, coronal, and axial MRI images, positioned at MNI co-ordinates [-40, -40, +10] which are medial to the pSTs area reported at [-63, -37, +6] by Wise et al. (2001). Cortical damage in the temporal lobe (at *z* = +8) was observed in 5/6 patients but only 1/6 patients had damage to Spt (at *z* = +20). There were no instances of Spt damage in the context of preserved temporo-parietal white matter tracts. However, there were three patients who had damage to the white matter but not to the more lateral cortical regions. Therefore, our results show that temporo-parietal white matter damage, in the vicinity of the arcuate fasciculus, was sufficient to cause selective auditory repetition difficulties but we do not know if selective damage to Spt would also cause auditory repetition difficulties.

The remaining 2/8 patients (including the patient with selective difficulty repeating non-words) had damage to a more anterior component of the superior longitudinal fasciculus at the level of the motor cortex (*y* = -10 in MNI space).

## DISCUSSION

The aim of this paper was to investigate the neurological underpinnings of non-semantically mediated sensory-to-motor translation during auditory repetition. On the basis of prior literature, we tested two hypotheses. The first was that a functionally defined area (Spt) in the TPJ would respond proportionally to the demands on non-semantically mediated auditory input-to-vocal tract output. This was based on prior fMRI data (Pa and Hickok, 2008; Hickok et al., 2009), and tested with a new fMRI experiment that aimed to systematically tease apart activation related to auditory processing and articulation from activation related to semantically and non-semantically mediated sensory-to-motor integration. The second hypothesis was that selective deficits in translating auditory inputs to motor outputs during auditory repetition, when auditory comprehension and speech production were preserved (i.e., conduction aphasia), would be the consequence of damage to the arcuate fasciculus. This was based on the classic neurological model of language, where the arcuate fasciculus functions to connect auditory images of speech in Wernicke’s area to motor images of speech in Broca’s area (Geschwind, 1965). As discussed below, we found evidence for the second but not for the first hypothesis.

Evidence in support of the arcuate fasciculus being essential for auditory-to-motor integration during auditory repetition was provided by a lesion analysis which considered whether selective deficits in auditory repetition in patients who had preserved auditory comprehension, picture naming, and verbal fluency were the consequence of lesions to: (1) TPJ/Spt with minimal involvement of the underlying white matter; (2) the temporo-parietal white matter tracts (in the vicinity of the arcuate fasciculus) with minimal involvement of TPJ/Spt cortex; (3) both TPJ/Spt and the underlying white matter; or (4) neither TPJ/Spt nor the underlying white matter. The results from eight different patients provided consistent evidence (8/8) that selective difficulties with auditory repetition were the consequence of damage to white matter in the vicinity of the arcuate fasciculus. In 6/8 patients this was observed posteriorly in the temporal lobe, undercutting the left posterior superior temporal area associated with phonological processing during speech production. In the other two patients, the white matter damage was more anterior.

Although all eight patients with selective deficits in auditory repetition had white matter damage in the vicinity of the arcuate fasciculus, only one had damage that extended into the cortex surrounding the peak MNI co-ordinates associated with Spt [-51, -39, +21] in our fMRI study. Thus the lesion results provide evidence that selective repetition difficulties can result from white matter damage in the vicinity of the arcuate fasciculus when Spt is intact, but we did not find evidence that selective repetition difficulties can be caused by damage to the cortical area Spt when the white matter tract is intact.

The distinction between cortical and white matter damage is not provided in the lesion analysis reported by Buchsbaum et al. (Buchsbaum et al., 2011) who show evidence that 12/14 of their patients with auditory repetition and picture naming difficulties had very extensive temporo-parietal damage that overlapped with the relatively small Spt cortical area identified in their fMRI experiment. In contrast, the lesion overlap was smaller in our patients who were selected to have focal lesions and deficits in auditory repetition but not picture naming, word comprehension, or verbal fluency. Our finding that some of our conduction aphasics had focal white matter damage that spared the surrounding gray matter (see bottom row of **Figure 4**) suggests new directions for neurocomputational models of aphasia (Ueno et al., 2011). For example, Ueno et al. (2011) use a connectionist neural network to model conduction aphasia both by subtracting incoming links (simulating white-matter damage) and by simultaneously adding noise to unit outputs (simulating gray matter damage), whereas our finding suggests that the white-matter damage alone may suffice. Furthermore, our finding that the maximum overlap of damage in 6/8 of our patients was at the level of the left posterior superior temporal sulcus also stands in contrast to previous suggestions for the involvement of the supramarginal gyrus (Ueno et al., 2011) or the supratemporal plane (Buchsbaum et al., 2011).

**FIGURE 5:**
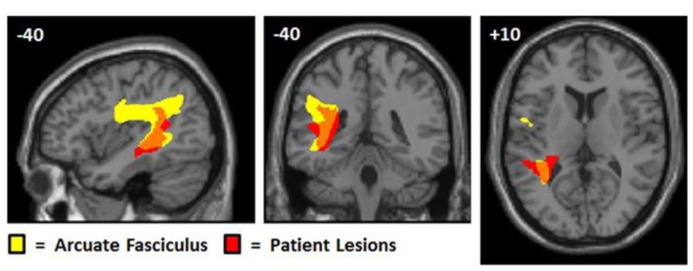
**Overlap between patient lesions and arculate fasciculus.** This figure shows the arcuate fasciculus (yellow) from Ffytche et al.’s (2010) probabilistic atlas with the lesion overlap map for our patients, positioned at MNI co-ordinates (-40, -40, +10). The probabilistic tractography image was thresholded at 0.25 and the lesion overlap map was thresholded to show areas where damage overlapped for at least four patients. Although the lesion effects are consistent with selective deficits in repetition arising from damage to the white matter connections linking temporal and frontal areas, we cannot say, with any precision, which cortical regions have been disconnected. The lesions are likely to damage the multiple tracts that overlap in this area connecting both temporal and parietal regions to different motor and prefrontal regions (Ffytche et al., 2010).

The lesion analysis is consistent with the importance of the arcuate fasciculus for auditory repetition (see **Figure 5**). In our sample of patients, we found no evidence for or against the importance of Spt in sensory-to-motor integration. For this we would need to find patients with selective damage to Spt who had minimal involvement of the underlying temporo-parietal white matter. Such damage would be highly unlikely following a stroke because of the underlying vascular anatomy. We turn now to our fMRI experiment, which set out to investigate whether activation in a functionally identified Spt area was sensitive to the demands on auditory-to-motor integration when auditory input and the demands on articulation were tested independently.

If Spt is involved in non-semantic auditory-to-motor integration then we would expect activation to be higher for auditory repetition of pseudowords than for naming the source of nonverbal sounds, where the motor response is (a) semantically mediated and (b) does not mimic the auditory input. In contrast, we found that Spt activation was higher for naming nonverbal sounds than repetition of words or pseudowords. Prior literature does not suggest that Spt is selectively involved in semantically mediated sensory-motor integration because Spt activation has been reported for humming music (Pa and Hickok, 2008). Likewise, our study found no evidence that Spt activation increased for semantically mediated sensory-motor integration. In fMRI Paradigm 1, we found that Spt activation was lower for semantically mediated speech production during (i) word repetition relative to pseudoword repetition and (ii) object naming relative to pseudoword reading. In fMRI Paradigm 2, Spt activation during picture naming (semantically mediated sensory-to-motor integration) did not differ from that during an articulation task that involved no sensory-to-motor integration (saying “1-2-3” to the same pictures).

The pattern of activation in Spt is also inconsistent with what would be expected from the motor control of articulation, because we would expect the demands on articulatory planning to increase with novelty (pseudowords relative to words) and not to differ when the articulatory output was matched across participants (object naming versus reading in both fMRI Paradigms 1 and 2). Strikingly, however, the pattern of activation in Spt is consistent with that associated with auditory processing in response to auditory stimuli (greatest for nonverbal sounds irrespective of task), auditory feedback from the sound of the spoken response (speech production relative to one-back task). Indeed, the response pattern in Spt was very similar to that observed in the primary auditory cortex (left Heschl’s gyrus), the main difference being that left Heschl’s gyrus did not respond during the one-back task on visual pseudowords (fMRI Paradigm 1), nor did it respond during semantic decisions on written words (fMRI Paradigm 2). Thus, Spt distinguishes itself from primary auditory cortex because it appears to be an auditory site that is activated in conditions that might generate auditory associations in the absence of auditory stimuli. Such a conclusion is consistent with many prior studies that have reported TPJ activation during tasks that involve auditory imagery or auditory short term memory (Paus et al., 1996a,b; Shergill et al., 2001; Wise et al., 2001; Buchsbaum and D’Esposito, 2009; Koelsch et al., 2009; McGettigan et al., 2011). In brief, we are proposing that TPJ/Spt activation during covert rehearsal of auditory words (Hickok et al., 2009) reflects internal representations of sounds (akin to auditory imagery). This may be involved in, and even contribute to, articulatory planning, irrespective of how speech production is driven (e.g., sensory inputs, object recognition, counting or verbal fluency). The role of auditory imagery in speech production therefore contrasts to what is implied by the term “sensory motor integration” in which the motor output is computed from the sensory input. We cannot unpack all the different cognitive and neural mechanisms that might be involved in speech production or integrate all the different labels and terminologies that have been used. Instead, we focus on our empirical results from this study where we investigated whether there are any brain areas that are more activated for non-semantically driven auditory-to-motor translation (i.e., during auditory pseudoword repetition) than semantically mediated auditory to motor processing (i.e., during nonverbal sound naming). We hypothesized that TPJ/Spt might be involved but we found no evidence to support this hypothesis. Instead, we suggest that non-semantically mediated auditory repetition may be supported by white-matter connections between auditory and motor areas (rather than in a cortical area that translates auditory to motor processing). Our findings allow us to provide further information about the functional response properties of the area commonly known as Spt.

Unraveling our own data, we are proposing that Spt activation is observed during (1) one-back matching of visual pseudowords because participants generate internal representations of the sounds associated with the pseudowords (i.e., their phonology); (2) semantic decisions on written words because as proposed by Glaser and Glaser (1989), participants access the sounds of words (i.e., phonology) when making semantic decisions; and (3) all overt articulation conditions because participants generate speech sounds that are processed in the auditory cortex and beyond. One might argue that auditory processing during articulatory activity could loosely be defined as sensory-motor processing. However, we have not found evidence that Spt/TPJ is required to transform sensory inputs to motor outputs. Therefore, it is unlikely to be an “integration” area. Instead, we are claiming that Spt/TPJ might reflect sensory processing after motor output which may or may not be fed back to influence the motor output.

Although we are arguing against a specific role for Spt in translating externally presented auditory stimuli into vocal tract gestures, it remains possible that auditory processing in Spt plays an important role in correcting speech production at a post articulatory stage, perhaps by matching auditory imagery of the expected spoken response with auditory processing of the generated spoken response and then relaying corrective signals to the motor cortex. Within a predictive coding framework (Friston and Kiebel, 2009), expectations are modeled within a cortical hierarchy as top-down (or “backwards”) connections to sensory processing regions, whereas the opposite bottom-up (or “forward”) connections to higher-order predictive regions represent error-propagation which applies when the top-down predictions are not adequate to match the sensory input. A similar matching process is associated with auditory error cells in the DIVA model of speech production (see, Guenther et al. (2006) for a formal description) which has suggested that these auditory error cells are located in Spt (Guenther and Vladusich, 2012). Support for this hypothesis comes from both behavioral and fMRI data. At the behavioral level, the importance of auditory feedback during speech production has been established in many prior experiments, for example showing that speech fluency is disrupted by delayed auditory feedback of one’s own voice (Stuart et al., 2002) and showing rapid compensation of speech when the pitch of the auditory feedback is shifted (Burnett et al., 1997; Houde and Jordan, 1998). At the neural level, several prior studies have shown that altered auditory feedback increases activation in the posterior planum temporal region relative to unaltered speech (Tourville et al., 2008; Zheng, 2009). The co-ordinates of this effect [i.e., (-66, -38, +22) in Tourville et al., 2008; (-66,-45, +15) in Zheng, 2009] are close to those associated with Spt (-50, -40, +20) although future studies are required to show that the location of the auditory feedback effect corresponds exactly to a functionally identified Spt.

In conclusion, neither the results from our fMRI nor lesion experiments were consistent with Spt functioning as an area that is required to actively integrate auditory inputs into vocal tract gestures. The fMRI data were more consistent with an account where Spt is highly responsive to bottom-up auditory processing, with weaker responses to internally generated sounds. Such activity does not necessarily drive the motor response even when it co-occurs with auditory-to-motor integration. In contrast, the lesion data provided clear evidence that the temporo-parietal white matter that connects the left posterior superior temporal sulcus to the motor cortex is needed for auditory-to-motor integration but not for word comprehension or speech production during picture naming or verbal fluency. This is consistent with the neurological tradition that has attributed conduction aphasia to damage to a white matter tract – the arcuate fasciculus – which connects the two major language centers, Wernicke’s area and Broca’s area (Geschwind, 1965).

## Conflict of Interest Statement

The authors declare that the research was conducted in the absence of any commercial or financial relationships that could be construed as a potential conflict of interest.
